# Left Sided Appendix without Visceral Tranposition

**Published:** 1914-05

**Authors:** 


LEFT SIDED APPENDIX WITHOUT VISCERAL TRANPOSITION. Chas. B. G. de Nancrede, of Ann Arbor, *Jour Mich. State Med. So.,* January, 1914, reports a case, shows several illustrations from museum specimens and summarizes the explanation as follows:

About the close of the tenth week of gestation the somewhat left sided practically straight intestinal tube has assumed an U-form and the cecal bud and the rudiments of the appendix are recognizable, and the cecum occupies approximately the umbilical region. About the close of the fourth month the cecum reaches its usual prenatal position beneath the right lobe of the liver by a process of so-called rotation, passing in front of and across the superior mesenteric artery and duodenum, subsequently to descend into the right iliac fossa, this descent occurring, according to some observers about the sixth month of intrauterine life; according to others this may not occur normally until after birth.

While the few photographs presented by no means exhaust the possible abnormalities of location and course of the colon, they should prove adequate for the present purposes. Numerous still more complicated and puzzling possibilities can obtain, induced by the traction of inflammatory adhesions alone, or from the effect of these, superadded to congenital variations; with such we cannot at present concern ourselves.

